# Implication of the hepatokine, fibrinogen-like protein 1 in liver diseases, metabolic disorders and cancer: The need to harness its full potential

**DOI:** 10.7150/ijbs.66834

**Published:** 2022-01-01

**Authors:** Xi-Hua Liu, Lian-Wen Qi, Raphael N. Alolga, Qun Liu

**Affiliations:** 1 State Key Laboratory of Natural Medicines, School of Traditional Chinese Pharmacy, China Pharmaceutical University, Nanjing 210009, China.; 2 Clinical Metabolomics Center, China Pharmaceutical University, Nanjing 211198, China.

**Keywords:** FGL1, liver diseases, metabolic disorders, cancer, immunotherapy

## Abstract

Fibrinogen-like protein 1 (FGL1) is a novel hepatokine that forms part of the fibrinogen superfamily. It is predominantly expressed in the liver under normal physiological conditions. When the liver is injured by external factors, such as chemical drugs and radiation, FGL1 acts as a protective factor to promote the growth of regenerated cells. However, elevated hepatic FGL1 under high fat conditions can cause lipid accumulation and inflammation, which in turn trigger the development of non-alcoholic fatty liver disease, diabetes, and obesity. FGL1 is also involved in the regulation of insulin resistance in adipose tissues and skeletal muscles as a means of communication between the liver and other tissues. In addition, the abnormally changed FGL1 levels in the plasma of cancer patients make it a potential predictor of cancer incidence in clinical practice. FGL1 was recently identified as a major functional ligand of the immune inhibitory receptor, lymphocyte-activation gene 3 (LAG3), thus making it a promising target for cancer immunotherapy except for the classical programmed cell death protein 1/programmed cell death ligand 1 (PD-1/PD-L1) axis. Despite the potential of FGL1 as a new cancer biomarker and therapeutic target, there are few related studies and much of what has been reported are superficial and lack depth and particularity. Therefore, elucidating the role and underlying mechanisms of FGL1 could be crucial for the development of promising diagnostic and therapeutic strategies for related diseases. Here, we provide a comprehensive review of the cellular mechanisms and clinical prospects of FGL1 in the prevention and treatment of liver diseases, metabolic disorders and cancer, and proffer suggestions for future studies.

## Background

Fibrinogen-like protein 1 (FGL1), also called hepatocyte-derived fibrinogen-related protein 1 (HFREP-1) or hepassocin (HPS), belongs to the fibrinogen family with mitochondrial promoter activity. FGL1 is a 68 kD homodimer protein consisting of two 34 kD disulfide bonds, and about 312 amino acids 1, 2. The genetic code is located on chromosome 8p22-21.3 in the human genome 3. FGL1 was initially considered as a significantly changed protein in hepatoma cells and subsequently cloned from the human hepatocellular carcinoma (HCC) specimen cDNA library 4. Similar to fibrinogen, FGL1 has a hydrophobic leader peptide and a characteristic amino acid of the carboxyl terminus with β-and γ-subunits 4. However, due to the lack of platelet-binding sites, cross-linking sites, and thrombin-sensitive sites that are required for blood clotting, FGL1 does not exhibit the classic coagulation function which is characteristic of other fibrinogen members 4.

FGL1 is mainly synthesized in parenchymal hepatocytes, but not in endothelial cells 1. With respect to its distribution in various tissues, dot blot analysis of human tissues showed that FGL1 was strongly expressed in adult liver, weakly expressed in the pancreas, and not expressed in other tissues 2. Subsequently, FGL1 was also detected in the brown and white adipose tissues of mice, mediating the cross-talk between an injured liver and adipose tissues 5. As a secretory protein, FGL1 also exists in the bloodstream 6. It is reported that approximately 20% FGL1 in plasma is in a free unbound state 7, an indication that the biological function(s) of FGL1 extend(s) far beyond the hepatocytes. Currently, Oncomine database and Lung Cancer Genome Mapping database have shown that FGL1 mRNA is upregulated in lung cancer, prostate cancer, melanoma, and colorectal cancer tissues, but downregulated in pancreatic cancer, liver cancer and head and neck cancer, thereby indicating the involvement of FGL1 in these pathological states 8.

Several studies have suggested the crucial involvement of FGL1 in liver regeneration, cell proliferation, glucose and lipid metabolism, and immune activation. As a liver protective factor, FGL1 accelerates the growth of liver cells by promoting mitochondrial mitosis in the event of liver injury. As a novel functional ligand of lymphocyte-activation gene 3 (LAG3), FGL1 can promote the proliferation of tumor cells 8. Effective inhibition of the FGL1/LAG3 axis can activate tumor T cell immunity, which provides a meaningful direction for tumor immunotherapy. Additionally, FGL1 appears to be an atypical biomarker for nonalcoholic fatty liver disease, type 2 diabetes and obesity, and is involved in regulation of adipogenesis, gluconeogenesis, and insulin resistance through diverse molecular mechanisms 9. We discuss here, the role of FGL1 in liver diseases, metabolic disorders and cancer. We also highlight how these functions could be harnessed for optimum therapeutic outcome.

## Role of FGL1 in liver diseases

### FGL1 in liver injury

The liver is an organ with remarkable regenerative ability. A healthy liver can initiate and regulate self-regeneration through an interplay between several cytokines and growth factors secreted by itself or other organs after resection or moderate injury 10, 11. FGL1 was found to be specifically upregulated during liver regeneration after damage by D-galactose, and exerts it effects by promoting DNA synthesis 1. Human FGL1 (hFGL1) corresponding to rat FGL1 was cloned and demonstrated to stimulate cell uptake of ^3^H-thymine and increased DNA synthesis in various primary animal hepatocytes, indicating the role of hFGL1 in promoting cell proliferation 2. Following the isolation of mouse fibrinogen-related protein-1 (MFREP-1), a mouse homologue of hFGL1, MFREP-1 mRNA was found to be significantly increased after 70% liver resection in mice, suggesting the role of FGL1 in regulating cell growth during liver regeneration 12. As a potential therapeutic target for fulminant hepatic failure, administration of FGL1 to rats can alleviate the degree of injury and mortality rate in D-galactose and CCl_4_-induced liver injury 13. Conversely, silencing of FGL1* in vivo* can aggravate D-galactose-induced liver injury 13. Besides chemical-induced damage, hepatic and plasma FGL1 was also elevated in response to radiation-induced liver injury 14. In brief, FGL1 exerts a net expansionary effect on cell growth during liver regeneration as a liver-specific mitogenic growth factor.

Mechanism-wise, it was discovered that the point of action of FGL1 in promoting liver regeneration is mainly between 784 bp-614 bp in the luciferase-FGL1 promoter. The transcription factors, signal transducer and activator of transcription (STAT3) and hepatocyte nuclear factor 1 (HNF1) controlled the hepatocyte-specific FGL1 transcription after hepatectomy 15. Moreover, FGL1-specific receptor exists on the membranes of the human hepatic cell line L02, and FGL1 induces phosphorylation of extracellular signal-regulated kinase 1/2 (ERK1/2) through the autocrine route to promote the proliferation of hepatocytes 16. Further mechanistic exploration revealed the activation of the epidermal growth factor receptor (EGFR) as an important step in FGL1-induced ERK signaling 17. In hyperglycemic crisis, high glucose increase STAT3 and protein phosphatase 2A (PP2A)-HNF1 activity, and further induced FGL1 expression 18. Therefore, FGL1 can ameliorate streptozotocin-induced hyperglycotoxicity in mice by increasing the body's antioxidant capacity 18.

In conclusion, FGL1 contributes to the self-repair process of an injured liver. When the liver is exposed to chemical drugs, radiation or hyperglycemic crisis, FGL1 levels are significantly elevated, a process that may depend on the binding of transcription factor STAT3 and HNF1 in the FGL1 gene promoter. Furthermore, FGL1 binds to the membrane-specific receptor of hepatocytes and induces cell proliferation through an autocrine mechanism, which is dependent on the EGFR/ERK/tyrosine protein kinase (Src) pathway. In addition, liver injury also enhances the expression of FGL1 in brown adipose tissues suggesting a cross-talk between the injured liver and adipose tissues 5.

### FGL1 in nonalcoholic fatty liver disease

Nonalcoholic fatty liver disease (NAFLD) is a leading cause of liver-related morbidity and mortality 19. Hepatocytes secrete ~560 hepatokines, many of which affect metabolism both locally and in distant organs and are linked to the pathogenesis of metabolic diseases 20, 21. A total of 393 subjects with (n = 194) or without (n = 199) NAFLD were enrolled in a study cohort to evaluate the serum FGL1 concentration. After an overnight 12-h fast, all subjects received biochemical blood tests. NAFLD subjects had a significantly higher serum FGL1 level than those without NAFLD (7699.7 ± 194.9 *vs*. 6112.3 ± 143.7 lg/ml;* p* <0.001) 22. In addition, serum FGL1 concentrations were elevated in diabetic patients with NAFLD (n = 50) compared with the diabetic group (n = 50) 23. FGL1 was significantly increased in high fat diet (HFD)-fed mice and primary hepatocytes treated with oleic acid, both of which constituted the *in vivo* and *in vitro* models of NAFLD 22. FGL1 silencing or overexpression consequently alleviated or worsened NAFLD as evinced by indices such as serum transaminase, triglyceride levels, inflammatory markers and lipid synthesis-related genes (sterol regulatory element-binding protein-1, fatty acid synthase and acetyl-CoA carboxylase-1) 22. Mechanistically, there are potential binding sites of STAT3 in the FGL1 gene promoter, and unsaturated fatty acid activates STAT3 to increase FGL1 expression. The blockade of STAT3 dose- dependently inhibited unsaturated fatty acid induced FGL1 expression, which accounts for the increased level of FGL1 under proinflammatory conditions 24. In addition, FGL1 regulates NAFLD through an ERK1/2-dependent pathway 22. FGL1 affects the phosphorylation of ERK1/2 to increase the activity of lipid synthesis-related genes and further induces lipogenesis. In contrast, PD98059, the ERK1/2 inhibitor, blocked FGL1-induced synthesis-related genes expression *in vitro* 22.

On the basis of the aforementioned, FGL1 may be an acute reactant of liver steatosis stress, and participates in hepatic lipid metabolism in an ERK1/2-dependent manner. FGL1 can therefore serve as a biomarker candidate in the pathogenesis of NAFLD and as an indicator of treatment response.

## Role of FGL1 in metabolic diseases

### FGL1 in type 2 diabetes

It was reported that plasma FGL1 levels in diabetic patients were significantly higher than the normal population, but multiple linear regression analysis showed that the elevated FGL1 was independently associated with fasting glucose levels, insulin resistance, impaired fasting glucose, impaired glucose tolerance and newly diagnosed diabetes 25, 26. FGL1 null mice exhibited fasting hyperglycemia and enhanced hepatic glucose production 25. FGL1 plays a crucial role in the development of insulin resistance by regulating ERK1/2 activity, indicating that it may be a risk factor for prediabetes, and could be a potential diagnostic marker of diabetes 25.

The occurrence of diabetes is closely related to NAFLD. A recent meta-analysis has shown that NAFLD increases the risk of type 2 diabetes by about 2-fold 27. The hepatokines could be therapeutic targets for both NAFLD and diabetes 28. The relationship between FGL1 and liver steatosis in diabetic patients with or without NAFLD was investigated and a significant increase of serum FGL1 in type 2 diabetics and NAFLD patients, providing evidence that FGL1 elevation in both diseased conditions may facilitate hepatic lipid accumulation 28.

### FGL1 in obesity

Obesity is the consequence of metabolic disorders in multiple organs 29. The liver plays a central role in regulating energy balance by sensing nutrient availability and altering metabolite or energy production from various organ systems, including the central nervous system, adipose tissues, and skeletal muscles. The secretory hepatokines are critical in influencing metabolic phenotypes through inter-organ communication 30. Recently, the relationship between FGL1 and obesity was confirmed in obese individuals. The plasma FGL1 of obese subjects was higher than that of healthy individuals, and univariate analysis confirmed that BMI, waist circumference, total fat, visceral fat, and subcutaneous fat area were all positively correlated with FGL1 level 31. FGL1 null mice were shown to have abnormal plasma lipid profiles, fasting hyperglycemia and exhibited differences in white and brown adipose tissue morphology 5. Mechanism-wise, Jeong *et al.* found that palmitate induced FGL1 expression in primary hepatocytes through endoplasmic reticulum (ER) stress and p38-mediated pathway, and also triggered the transcriptional activation of FGL1 via binding of CCAAT/enhancer-binding protein β (C/EBPβ) to its promoter 32. Skeletal muscle is the main site of glucose utilization. Jeong* et al.* found that FGL1 mainly responded to hyperlipidemic environment through the phosphorylation of c-Jun N-terminal kinase (JNK), and further impaired insulin sensitivity through EGFR-mediated pathway, but did not affect inflammatory response and ER stress in skeletal muscles 32.

FGL1 is a communication factor that transmits signals of liver metabolic disorders to other tissues. Changes in FGL1 levels in the liver not only lead to hepatic metabolic disorders, but also affect obesity-related insulin resistance in adipose tissues and skeletal muscles. As a potential diagnostic biomarker and indicator of NAFLD, diabetes and obesity treatment response, FGL1 is a candidate target worthy of further study at the mechanistic level in the continual search for therapeutic remedies for these diseases.

## Role of FGL1 in cancer

### FGL1 in liver cancer

FGL1 is a highly expressed protective hepatocyte mitogen in the liver after resection. Studies have shown that FGL1 is underexpressed in hepatocellular carcinoma (HCC) at both the protein and mRNA levels 3. Mice that lack FGL1 develop HCC at a rate more than twice that of wild type mice treated with diethyl nitrosamine. Consistent with these findings, hepatocellular cancers from FGL1 knockout mice grew faster than those from the wild type mice 33. Furthermore, the level of FGL1 in HCC was found to be linked to the degree of cell differentiation, as poorly differentiated HCCs tend to have lower levels of FGL1 compared to highly differentiated HCCs 3.

The possible reasons for the persistent low levels of FGL1 in the development of liver cancer have also been provided by some groups. Heterozygosity loss analysis of liver cancer specimen showed that 57.1% of HCCs have allele loss of the FGL1 gene on chromosome 8p22 3, a position associated with HCC progression and recurrence for frequently lost isopet genes 34. HNF1α acts as an important liver-specific *cis*-acting element for FGL1, and downregulation of HNF1α might responsible for the inhibition of FGL1 transcription in liver cancer 15. Mechanismwise, endogenous HNF1α binds to high mobility group box-1 protein (HMGB1) and cAMP-response element binding protein (CREB), and activates FGL1 promoter by interacting with the 470-457 bp fragment of the FGL1 upstream promoter 15. FGL1 also acts as a tumor suppressor in HCC through protein kinase B/mammalian target of rapamycin (Akt/mTOR) signaling 33, a critical pathway in the development and progression of HCC 35.

Interestingly, the paradoxical role of FGL1 in normal and cancer cells proliferation were partly explained by Cao *et al.* They showed that there is a signal-peptide dependent pattern for FGLI in liver-derived cells. It acts as a key positive regulator in non-tumor cells via autocrine signaling and a tumor suppressor in HCC cells through intracrine signaling 16.

### FGL1 in gastric, lung and other cancers

FGL1 was reported to be up-regulated in gastric cancer (GC) tissues and the survival time of GC patients with high FGL1 levels was markedly shorter than the patients with low FGL1 levels 36. Hence, high expression of FGL1 can be an independent predictor of poor prognosis for GC patients 36.

In addition, FGL1 is involved in the epithelial-mesenchymal transformation (EMT), which is activated in the malignant progression of cancer and plays an important role in cancer development 37. The most common type of lung cancer is non-small cell lung cancer (NSCLC), which accounts for about 85% of lung cancer cases 38. Liver kinase B1 (LKB1) controls the initiation, differentiation and metastasis of lung cancer cells, and is a key barrier to the development and progression of lung cancer 39. FGL1 was found to be significantly increased in LKB1 mutated lung adenocarcinoma 40. Silencing FGL1 was found to be a novel approach for the treatment of LKB1 mutated lung adenocarcinoma by inducing EMT and angiogenesis 40. EGFR, which is expressed in more than 60% of NSCLC, has become an important target for the treatment of NSCLC 41. FGL1 was also significantly upregulated in EGFR mutant NSCLC cells. Gefitinib is the first-line treatment for patients with advanced NSCLC with activating EGFR mutation. FGL1 was found to be highly expressed in gefitinib-resistant NSCLC cell line 42. The decreased signal for poly(ADP-ribose) polymerase 1 (PARP1)-caspase 3 pathway induced by silencing FGL1 may be the underlying mechanism for gefitinib-resistant cell apoptosis 42.

Wang *et al.* conducted a meta-analysis of the Oncomine databases to map the level of FGL1 in a series of solid tumors, and found FGL1 is highly expressed in lung cancer, prostate cancer, breast cancer, melanoma and colorectal cancer, but downregulated in pancreas cancer, head and neck cancers 8.

### FGL1 in cancer immunotherapy

Immunotherapy is a type of cancer treatment that helps the immune system to fight cancer. Immune checkpoint blockade therapy marked the beginning of a new era of immunotherapy. It uses monoclonal antibodies to directly target the antigens, and block the pathways involved in T cell inhibition, such as cytotoxic T lymphocyte antigen-4 (CTLA4) and programmed cell death protein 1 (PD-1) 43. LAG3 is a promising next-generation immune checkpoint and its blockade is actively pursued in clinical trials 44. As a receptor, LAG3 can transmit immunosuppressive signals and negatively regulate the proliferation and activation of CD4+ and CD8+ T cells 44. The emergence of FGL1 as a major functional ligand of LAG3 has attracted great attention in the field of tumor immunotherapy. FGL1 was found strongly binds to LAG3 on the surface of T cells, and the interaction of these two proteins is independent of MHC class II, a canonical ligand of LAG3 8. Tumor growth in LAG3 or FGL1 knockout mice was suppressed after colon cancer cells transplantation and by further blocking the interaction between FGL1 and LAG3, stimulated the activation and proliferation of T cells in the tumor microenvironment, thus improving immunity and the elimination of tumors 8.

Blockade of the FGL1/LAG3 pathway has been exploited by many a researcher as an immunotherapeutic route for cancers. FGL1/LAG3 blockade combined with natural products, seems to be more effective against liver cancer. Oxysophocarpine is a known alkaloid with demonstrable anti-HCC properties both *in vivo* and *in vitro* by reducing FGL1 expression and sensitizing CD8+ T cells to LAG3 immunotherapy 45. Blockade of the FGL1/LAG3 axis with concurrent administration of metformin can enhance T-cell-mediated immune response, improve the tumor microenvironment immunosuppression, and enhance general anti-tumor immunity 46.

NSCLC patients with high FGL1 expression have poor outcomes when treated with PD-1/programmed cell death ligand 1 (PD-L1) inhibitors. Intriguingly, when FGL1/LAG3 and PD-1/PD-L1 axes were simultaneously inhibited, the therapeutic effect was significantly improved, evidenced by a longer lifespan and reduced tumor burden in mice 42. In addition, the combination of PD-1/PD-L1 signaling blockade and FGL1 gene silencing showed a high synergistic effect in the treatment of breast cancer 46. Together, the FGL1/LAG3 and PD-1/PD-L1 axes might be independently targeted in treating cancers though synergism of the two yields better therapeutic outcomes.

## Role of FGL1 in rheumatoid arthritis

Rheumatoid arthritis (RA) is a common, chronic, systemic autoimmune inflammatory disease. Early diagnosis of RA is crucial in combating the long term irreversible pathophysiological damage that would otherwise occur 47. In a proteomic study involving 1244 clinical samples, FGL1 levels were 10-time higher in RA patients than in healthy subjects, and acted as one of the most differentially expressed immune/inflammation-related proteins in RA patients with moderate and high disease activity 48. Thus, FGL1 has emerged as a novel protein capable of predicting disease progression in RA patients before and after treatment.

## Conclusions and future directions

### Harnessing the potential of FGL1 as a hepatokine

**Figure 1:**
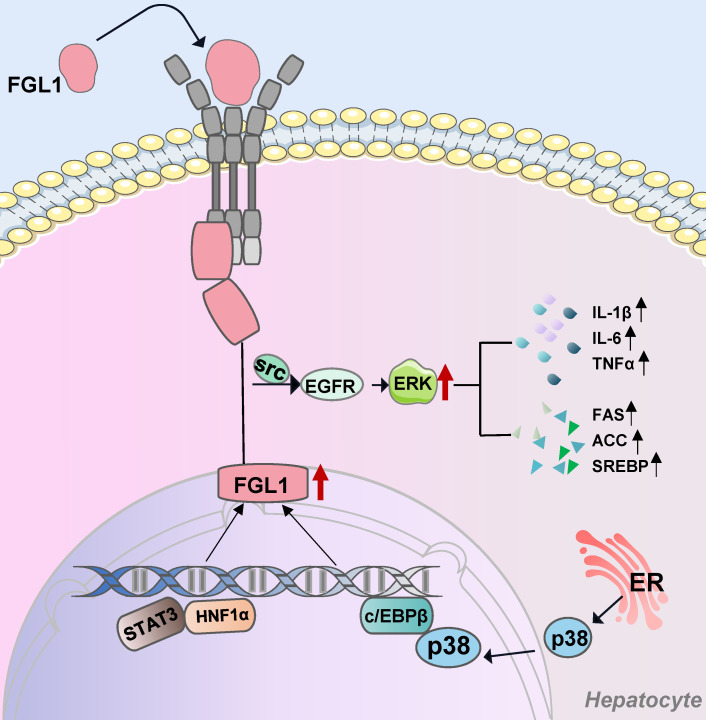
** FGL1-involved signaling pathways in hepatocytes.** Extracellular FGL1 binds to specific hepatocytes membrane receptors and enters the cells. Transcription factors STAT3, HNF1α, and C/EBPβ bind to the intracellular FGL1 promoter region to promote its transcriptional activation. Elevated FGL1 exerts its biological functions through the EGFR/SRC/ERK cascade. FGL1: fibrinogen-like protein 1; Src: tyrosine protein kinase; EGFR: epidermal growth factor receptor; ERK: extracellular signal-regulated kinase; p38: mitogen-activated protein kinase; STAT3: signal transducer and activator of transcription 3; HNF1α: hepatocyte nuclear factor 1 alpha; c/EBPβ: CCAAT/enhancer-binding protein β; ER: endoplasmic reticulum.

**Figure 2:**
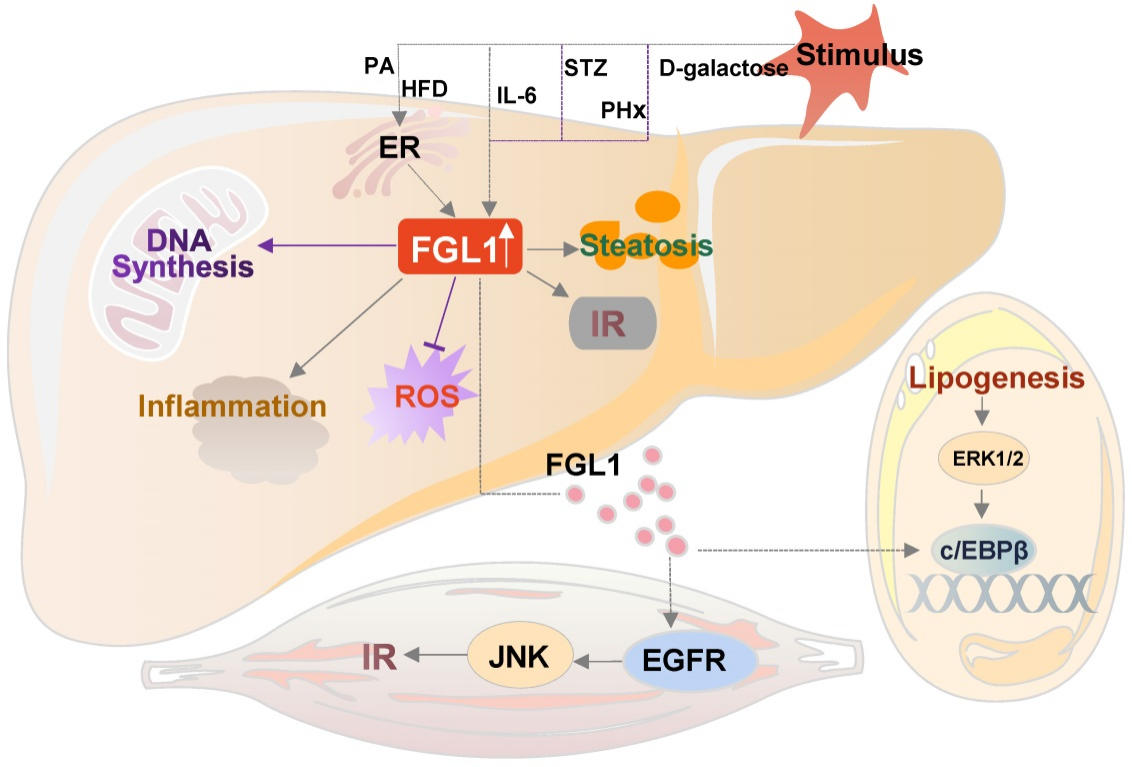
** The crosstalk of FGL1 in metabolic diseases.** Elevated FGL1 in the liver promotes DNA synthesis, inhibits ROS production, and causes insulin resistance, steatosis and inflammation. Excess FGL1 causes obesity-related insulin resistance in skeletal muscle through the EGFR/JNK-mediated pathway, and adipogenesis through an ERK1/2-c/EBPβ-dependent pathway in the adipose tissue. HFD: high-fat diet; PA: palmitic acid; IL-6: interleukin 6; STZ: streptozotocin; PHx: partial hepatectomy; ER: endoplasmic reticulum; FGL1: fibrinogen-like protein 1; c/EBPβ: CCAAT/enhancer-binding protein β; EGFR: epidermal growth factor receptor; ERK1/2: extracellular signal-regulated kinase1/2; IR: insulin resistance; JNK: c-Jun N-terminal kinase.

The liver, a key organ that regulates energy homeostasis in the human body, secretes proteins called hepatokines, which play crucial roles in diverse medical conditions such as insulin resistance 49. The biological roles of hepatokines in systemic communication between the liver and other organs is a rapidly developing field and could become a scientific hotspot 50,51. As a hepatokine, extracellular FGL1 binds to specific hepatocytes membrane receptors and enters the cells. Otherwise, transcription factors STAT3, HNF1α, and C/EBPβ bind to the intracellular FGL1 promoter region to promote its transcriptional activation. In addition, elevated FGL1 exerts its biological functions through the EGFR/Src/ERK cascade (**Figure 1**). FGL1 also acts as a transmitter of communication between the liver, skeletal muscles, and adipose tissues, as early on outlined and schematically summarized in **Figure 2**. In brief, elevated FGL1 in the liver under external stimulation promotes DNA synthesis, inhibits ROS production, and causes insulin resistance, steatosis and inflammation. Excess FGL1 also causes obesity-related insulin resistance in skeletal muscle through the EGFR/JNK mediated pathway. In addition, high levels of FGL1 induce adipogenesis through an ERK1/2-C/EBPβ-dependent pathway in adipocytes.

Taking due cognisance of the aforementioned cardinal pathways and the interplay that exists between them, the medical significance of the hepatokine, FGL1 cannot be overemphasized. There is therefore need to further explore the known as well as search for newer mechanistic pathways that can be harnessed for therapeutic benefits.

### Harnessing the prospects of FGL1 as a therapeutic target

Among the immunodetection sites discovered so far, the inhibitory effect of PD-1/PD-L1 on tumor development has been most thoroughly studied. Clinically, the effective rate of PD-1/PD-L1 inhibitors is approximately 80% in lymphoma, 60% in high microsatellite instability tumors, and 10% ~ 30% in other common solid tumors 52, thereby limiting its wide-scale applicability. Therefore, it is very necessary to search for immune escape mechanisms other than the PD-1/PD-L1 route. FGL1 has recently been identified as a ligand of LAG3, providing scientific evidence for same as a possible therapeutic target. The discovery of immunotherapy via the LAG3/FGL1 axis represents a milestone in cancer treatment. Patients who do not respond to PD-1/PD-L1 inhibitors may have access to alternative therapies. It would be worthwhile investigating the simultaneous blockade of both PD-1/PD-L1 and FGL1/LAG3 axes in further research.

As earlier indicated, FGL1 might be a vital marker in cancer initiation and progress. A summary of all reported FGL1-mediated effects in cancers is summarized in **Table 1**. More emphasis could be laid on monoclonal antibodies or small interfering RNA associated with FGL1, and/or as combination therapy with first-line drugs or natural products with proven anticancer properties as alternatives. These traditional remedies either used holistically or on a single-component basis, could be investigated in the context of further exploring FGL1 as the therapeutic target in different cancers.

**Figure 3:**
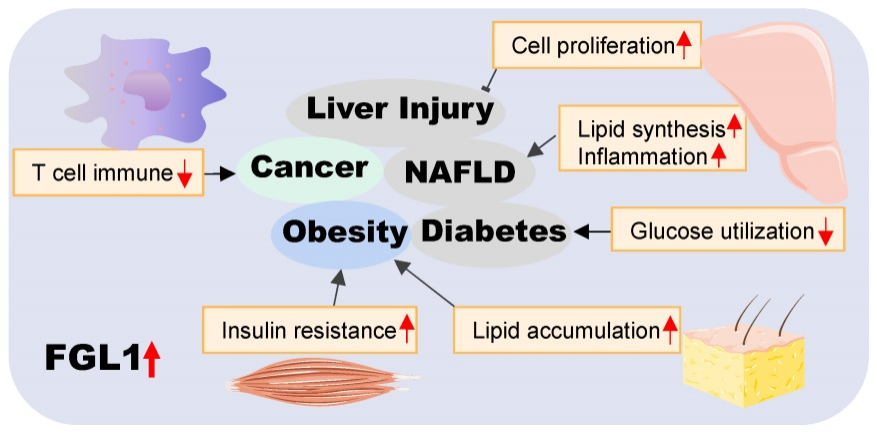
** Summary of the role of FGL1 in various diseases.** Elevated FGL1 regulates glucose and lipid metabolism, insulin resistance, cell proliferation, and immune response, leading to obesity, diabetes, non-alcoholic fatty liver disease, cancer and other diseases.

In conclusion, we have provided an overview of the roles of FGL1 in various diseases in this review (pictorially summarized in **Figure 3**). On the basis of available literature, FGL1 is vest with two key functional identities, as a hepatokine and a therapeutic target. The former plays a hepatoprotective role in liver regeneration and self-repair after injury, as well as a communicator between different organs in metabolic diseases. The latter identity presents FGL1 as a possible novel diagnostic biomarker or therapeutic target for cancers and various metabolic-associated diseases.

## Figures and Tables

**Table 1:** Functional roles of FGL1 in different types of cancer

Cancer types	FGL1 levels	Study subjects	Key messages	Ref.
Liver cancer	↓	Human solid tumors and human HCC cell lines	The level of FGL1 in HCC cells is closely related to tumor progression and degree of differentiation, and the suppression of FGL1 enhances HCC cell growth.	3
		Mouse xenotransplant	Knockout of FGL1 resulted in HCC cell proliferation, reducing the incidence of HCC in an Akt/mTOR dependent manner.	33
		HepG2 cells and mouse	Down-regulation of HNF1α causes, at least in part, the transcriptional down-regulation of FGL1 in HCC.	15
		Human solid tumors	Meta-analysis of the Oncomine databases revealed the downregulation of FGL1 mRNA in human liver cancer solid tumors.	8
Lung cancer	**↑**	Human solid tumors	Loss of FGL1 may be a novel approach to promote EMT and angiogenesis in patients with LKB1 mutant lung adenocarcinoma.	8
		PC9 or PC9/GR cells and mouse xenotransplant	Loss of FGL1 can improve the acquired resistance of gefitinib in NSCLC via PARP1/caspase 3 pathway.	42
		Human soild tumors	Meta-analysis of the Oncomine databases revealed the upregulation of FGL1 mRNA in human lung cancer solid tumors, with the highest percentage of upregulation (35%) in lung cancer datasets.	8
Gastric cancer	**↑**	Human solid tumors	FGL1 has the potential to be a predictor in GC patients as well as a target for the treatment of GC.	36
Breast cancer	**↑**	Mouse xenotransplant	Targeted delivery of siFGL1 and metformin can inhibit the growth and migration of breast cancer cells.	8
Prostate cancer	**↑**	Human solid tumors	Meta-analysis of the Oncomine databases and TCGA cancer database revealed the upregulation of FGL1 mRNA in human prostate cancer solid tumors.	8
Melanoma cancer	**↑**	Human solid tumors	Meta-analysis of the Oncomine databases revealed the upregulation of FGL1 mRNA in human melanoma solid tumors.	8
Colorectal cancer	**↑**	Human solid tumors	Meta-analysis of the Oncomine databases revealed the upregulation of FGL1 mRNA in human colorectal cancer solid tumors.	8
Pancreas cancer	**↓**	Human solid tumors	Meta-analysis of the Oncomine databases revealed the downregulation of FGL1 mRNA in human pancreas solid tumors, with 60% downregulation.	8
Head and neck cancers	**↓**	Human solid tumors	Meta-analysis of the Oncomine databases revealed the downregulation of FGL1 mRNA in human head and neck cancers solid tumors.	8

*Note*: Upward arrows represent up-regulation of FGL1 levels and the downward arrows represent down-regulation of FGL1 levels.

## Abbreviations

Akt: protein kinase B
c/EBPβ: CCAAT/enhancer-binding protein β
CREB: cAMP-response element binding protein
CTLA4: cytotoxic T lymphocyte antigen-4
ER: endoplasmic reticulum
EMT: epithelial-mesenchymal transformation
ERK1/2: extracellular signal-regulated kinase1/2
EGFR: epidermal growth factor receptor
FGL1: fibrinogen-like protein 1
GC: gastric cancer
HMGB1: high mobility group box-1 protein
HFREP-1: hepatocyte-derived fibrinogen-related protein 1
HNF1α: hepatocyte nuclear factor 1 alpha
HFD: high fat diet
HPS: hepassocin
HCC: human hepatocellular carcinoma
hFGL1: human FGL1
JNK: c-Jun N-terminal kinase
LAG3: lymphocyte-activation gene 3
LKB1: liver kinase B1
mTOR: mammalian target of rapamycin
MFREP-1: mouse fibrinogen-related protein-1
NSCLC: non-small cell lung cancer
NAFLD: nonalcoholic fatty liver disease
PARP1: poly (ADP-ribose) polymerase 1
PP2A: protein phosphatase 2A
PD-1: programmed death-1
PD-L1: programmed cell death ligand 1
RA: rheumatoid arthritis
STAT3: signal transducer and activator of transcription 3
Src: tyrosine protein kinase
